# Five novel clinical phenotypes for critically ill patients with mechanical ventilation in intensive care units: a retrospective and multi database study

**DOI:** 10.1186/s12931-020-01588-6

**Published:** 2020-12-10

**Authors:** Longxiang Su, Zhongheng Zhang, Fanglan Zheng, Pan Pan, Na Hong, Chun Liu, Jie He, Weiguo Zhu, Yun Long, Dawei Liu

**Affiliations:** 1 Department of Critical Care Medicine, State Key Laboratory of Complex Severe and Rare Diseases, Peking Union Medical College Hospital, Chinese Academy of Medical Science and Peking Union Medical College, Beijing, 100730 People’s Republic of China; 2 Department of Emergency Medicine, Sir Run Run Shaw Hospital, Zhejiang University School of Medicine, Hangzhou, 310016 People’s Republic of China; 3 Medical Data R&D Center, Digital China Health Technologies Co., Ltd., Beijing, 100080 People’s Republic of China; 4 Information Management Center, Peking Union Medical College Hospital, Peking Union Medical College and Chinese Academy of Medical Sciences, Beijing, 100730 People’s Republic of China; 5 College of Pulmonary and Critical Care Medicine, Chinese PLA General Hospital, Beijing, 100091 People’s Republic of China

**Keywords:** Mechanical ventilation, Critically ill patients, Machine learning, Clinical phenotype

## Abstract

**Background:**

Although protective mechanical ventilation (MV) has been used in a variety of applications, lung injury may occur in both patients with and without acute respiratory distress syndrome (ARDS). The purpose of this study is to use machine learning to identify clinical phenotypes for critically ill patients with MV in intensive care units (ICUs).

**Methods:**

A retrospective cohort study was conducted with 5013 patients who had undergone MV and treatment in the Department of Critical Care Medicine, Peking Union Medical College Hospital. Statistical and machine learning methods were used. All the data used in this study, including demographics, vital signs, circulation parameters and mechanical ventilator parameters, etc., were automatically extracted from the electronic health record (EHR) system. An external database, Medical Information Mart for Intensive Care III (MIMIC III), was used for validation.

**Results:**

Phenotypes were derived from a total of 4009 patients who underwent MV using a latent profile analysis of 22 variables. The associations between the phenotypes and disease severity and clinical outcomes were assessed. Another 1004 patients in the database were enrolled for validation. Of the five derived phenotypes, phenotype I was the most common subgroup (n = 2174; 54.2%) and was mostly composed of the postoperative population. Phenotype II (n = 480; 12.0%) led to the most severe conditions. Phenotype III (n = 241; 6.01%) was associated with high positive end-expiratory pressure (PEEP) and low mean airway pressure. Phenotype IV (n = 368; 9.18%) was associated with high driving pressure, and younger patients comprised a large proportion of the phenotype V group (n = 746; 18.6%). In addition, we found that the mortality rate of Phenotype IV was significantly higher than that of the other phenotypes. In this subgroup, the number of patients in the sequential organ failure assessment (SOFA) score segment (9,22] was 198, the number of deaths was 88, and the mortality rate was higher than 44%. However, the cumulative 28-day mortality of Phenotypes IV and II, which were 101 of 368 (27.4%) and 87 of 480 (18.1%) unique patients, respectively, was significantly higher than those of the other phenotypes. There were consistent phenotype distributions and differences in biomarker patterns by phenotype in the validation cohort, and external verification with MIMIC III further generated supportive results.

**Conclusions:**

Five clinical phenotypes were correlated with different disease severities and clinical outcomes, which suggested that these phenotypes may help in understanding heterogeneity in MV treatment effects.

## Background

Ventilator-associated lung injury (VALI) commonly occurs in patients hospitalized in the intensive care units (ICUs). A growing body of studies have shown that VALI is not limited to patients with acute respiratory distress syndrome (ARDS) but might occur in patients undergoing mechanical ventilation (MV) without lung injury; improper placement of the ventilator can also cause VALI [1, 2]. The population undergoing surgeries under anesthesia requiring short-term ventilation is a well-documented group. Each year, approximately 230 million patients worldwide are administered MV for surgical anesthesia. Most of these patients may have no basic lung disease before surgery, but after a short period of MV, the incidence of postoperative pulmonary complications has been shown to be 5% to 40%, and it has been shown to occur in patients undergoing chest or abdominal surgery in particular; these patients are more likely to develop postoperative edema, consolidation, and even ARDS [3]. As it is the most serious postoperative pulmonary complication, ARDS is the leading cause of postoperative respiratory failure [2, 3]. It is worth noting that in patients without lung injury who receive MV for more than 48 h, the incidence of ARDS within 5 days is nearly 20% [4]. Therefore, identifying distinct clinical phenotypes and adopting different ventilation strategies for different patients accordingly may potentially benefit both critical care clinical research and practices.

Previous clinical studies of MV in critically ill patients have analyzed MV as a homogeneous disease or only discussed how to perform pulmonary protective ventilation in patients with ARDS [5–7]. In addition, the current methods of selection, assessment, and intervention for MV in the ICU rarely consider distinct clinical phenotypes in patients receiving MV. However, distinct clinical phenotypes of MV are actually associated with different disease severities and prognoses and may be caused by a variety of underlying mechanisms. It is unclear whether various phenotypes of MV should be distinguished when clinicians administer MV treatments. There is no publicly available evidence showing whether clinicians should only focus on small tidal volume lung protection or a certain target, such as the plateau pressure, tidal volume (VT), or positive end-expiratory pressure (PEEP), when they treat critically ill patients. To resolve the practical problems encountered in the clinic and overcome the bottleneck of traditional studies, we used a database and machine learning based method to explore the significance of MV phenotypes. Perhaps there are some potential phenotypes relevant to disease severity and patient outcomes that may contribute to MV therapy. The model can predict which phenotype the patient is and guide the next step of treatment.

## Methods

### Patient data collection

Using the administrative database of Peking Union Medical College Hospital and the data from our previously published study in Critical Care Medicine, we conducted a retrospective study of patients undergoing MV [8]. From May 2013 to December 2016, we identified MV patients admitted to the ICU of Peking Union Medical College Hospital. Patients younger than 18 years of age or admitted to the ICU were excluded from the 24-h period. The Institute of Institutional Research and Ethics of Peking Union Medical College Hospital approved this study involving human subjects. We retrieved data on a total of 5013 unique adult individuals from Peking Union Medical College Hospital who received MV during their ICU stay. We randomly divided this batch of data into two groups according to a ratio of 4:1. A total of 4009 patients were enrolled as training cohort, and 1004 patients were collected as validation cohort.

### Ventilator mode selection

Lung-protective strategies for MV were performed on all of the patients who were admitted to the ICU. When the patients were under adequate sedation and analgesia but without spontaneous breathing, volume-controlled or pressure-controlled ventilation was used. Once the patient had spontaneous breathing, controlled ventilation was immediately converted to pressure support ventilation.

### Statistical methods

We selected 22 candidate variables based on their relevance to MV. These mainly included demographic characteristics (age and temperature (T)), scores (acute physiology and chronic health evaluation (APACHE) II scores and sequential organ failure assessment (SOFA) scores), respiratory variables (respiratory rate (RR), oxygen concentration in the inhalation gas (FiO_2_), pulse oxygen saturation (SpO_2_%), mean airway pressure (Pmean), peak airway pressure (Ppeak), positive end-expiratory pressure (PEEP), tidal volume (VT), partial pressure of oxygen (pO_2_) and partial pressure of carbon dioxide (pCO_2_)), circulatory and perfusion variables (heart rate (HR), mean arterial pressure (MAP), central venous pressure (CVP), static arterial carbon dioxide partial pressure (Pv-aCO_2_), lactate, P(v-a)CO_2_/C(a-v)O_2_ ratio, perfusion index (PI) and hemoglobin (Hb)) and a persistence variable (fluid balance). For each variable, we computed the mean value during the first 24 h of hospital presentation. To derive the phenotypes of MV, we first evaluate the distribution, missingness, and correlation of the above candidate variables. Due to the scarcity of the data samples, the missing variables were not simply eliminated. Instead, multiple imputation with chained equations was used to account for missing data. We also excluded highly correlated variables using rank-order statistics in the sensitivity analysis with threshold value of 0.75. Because the data type of the candidate variables was numerical, we used latent profile analysis (Gaussian mixture model) to recover hidden groups based on the means of the continuous variables observed. It always involved multiple constraint conditions: “model_1” involved equal variance and zero covariance, and “model_2” involved varying variance and zero covariance. An algorithm, the “expectation maximization” (EM) algorithm, was used to find the means and standard deviations of these distributions through two steps. First, we started with some initial starting guesses of the means and standard deviations. Based on these guesses, we assigned a posterior probability of being in a certain group to each patient. Second, these posterior probabilities were then used to update our guesses of the within-class parameters, which, in turn, were used to update the posterior probability, and so on until nothing seemed to change much anymore. Then, “model_1” was adopted in this manuscript. In the latent class analysis, the optimal phenotype number was confirmed using a combination of Bayesian information criteria, adequate size, high median probabilities of group membership with each phenotype, maximum entropy, and clinical features of hidden groups.

To visualize the optimal phenotype results and patterns in clinical variables, the data were analyzed with three types of plots: (1) ranked plots of the variables by the mean and standardized difference among the phenotypes, (2) t-distributed stochastic neighbor embedding (t-SNE) plots (which show multidimensional data in three dimensions), and (3) alluvial plots (which show the proportional distribution of phenotype members across specific variables).

Since the SOFA score is used to track a person's disease severity during their stay in the ICU and determine the extent of his or her organ function or rate of failure, we also performed an analysis to determine the relationship between the new phenotypic classification and the SOFA score, which included determining (1) whether the traditional SOFA score reflects the severity of the disease; (2) whether the phenotypes overlap with the SOFA score area on the chord graph; and (3) the deaths associated with each phenotype and the corresponding quartile of the SOFA score (especially in the area with overlapping phenotypes). The mortality rate of different phenotypes was significantly different across regions, which was evaluated a posteriori by a clustering phenotypic method.

### External database verification

To further verify the phenotyping results yielded from Peking Union Medical College Hospital cohort, we then used another open database for external verification. The selected database is Medical Information Mart for Intensive Care III (MIMIC-III) database, which is a large, freely available database comprising deidentified health-related data associated with over 40,000 patients who stayed in critical care units of the Beth Israel Deaconess Medical Center between 2001 and 2012. According to the same inclusion criteria, data from 9457 patients were enrolled in our external verification study.

## Results

### Five clinical MV phenotypes

Summary information on our patient population is shown in Table 1. Multiple imputation with chained equations was employed to fill null values for each variable. After data preprocessing, we used latent profile analysis to derive groups. It was found that the five-class model was the optimal fit, forming the probability density distributions (1),

1 $$p = \mathop \sum \limits_{l = 1}^{5} p_{l} Normal\left( {\mu_{l} ,\sigma } \right)$$

where $Normal\left({\mu }_{l},\sigma \right)$ is a Gaussian distribution with a mean value ${\mu }_{l}$ and variance $\sigma$. The resulting probability density weight, mean value and variance are shown in Eq. 2.2

**Table 1:** Summary statistics of selected features in each group

Features (mean ± sd, missing ratio)	Whole group (n = 4009)		Survivors (n = 3776)		Nonsurvivors (n = 233)		*P* value
Age (years)	58.92 ± 16.83	0.00%	58.85 ± 16.87	0.00%	60.03 ± 16.27	0.00%	2.88E−01
T (℃)	36.97 ± 0.58	0.00%	36.97 ± 0.57	0.00%	37.04 ± 0.78	0.00%	1.40E−01
APACHE2	15.3 ± 7.55	12.45%	14.5 ± 6.65	12.42%	28.31 ± 9.26	12.88%	1.00E−53
SOFA	6.64 ± 3.77	10.03%	6.3 ± 3.47	9.83%	12.45 ± 3.82	13.30%	1.00E−58
RR (insp/min)	23.07 ± 24.37	0.00%	21.16 ± 20.99	0.00%	54 ± 45.44	0.00%	6.68E−23
FiO_2_ (%)	39.11 ± 8.54	0.00%	38.43 ± 7.46	0.00%	50.12 ± 15.02	0.00%	1.38E−25
SpO_2_ (%)	98.77 ± 1.95	0.00%	98.92 ± 1.4	0.00%	96.46 ± 5.28	0.00%	1.70E−11
Pmean (cmH_2_O)	8.86 ± 2.05	0.00%	8.68 ± 1.8	0.00%	11.79 ± 3.33	0.00%	2.30E−33
Ppeak (cmH_2_O)	17.97 ± 3.97	0.00%	17.71 ± 3.72	0.00%	22.08 ± 5.39	0.00%	5.00E−27
PEEP (cmH_2_O)	7.07 ± 4.62	0.00%	6.89 ± 4.42	0.00%	10.08 ± 6.59	0.00%	2.01E−07
VT (mL/B)	422.8 ± 72.86	0.00%	422.94 ± 72.39	0.00%	420.6 ± 80.23	0.00%	6.65E−01
PO_2_ (mmHg)	136.08 ± 38.96	0.00%	137.23 ± 37.93	0.00%	117.59 ± 49.4	0.00%	8.60E−09
PCO_2_ (mmHg)	38.79 ± 5.2	0.00%	38.6 ± 4.84	0.00%	41.94 ± 8.68	0.00%	1.85E−08
HR (BPM)	81.84 ± 24.51	0.00%	82.73 ± 22.9	0.00%	67.38 ± 40.23	0.00%	2.47E−08
MAP (mmHg)	89.63 ± 11.32	0.00%	89.76 ± 11.26	0.00%	87.53 ± 12.15	0.00%	6.80E−3
CVP (mmHg)	8.43 ± 2.47	43.18%	8.27 ± 2.36	45.39%	9.95 ± 2.93	7.30%	1.78E−14
Pv-aCO_2_ (mmHg)	5.67 ± 2.44	45.02%	5.7 ± 2.33	47.30%	5.34 ± 3.22	8.15%	1.12E−01
Lac (mmol/L)	2.15 ± 1.9	0.00%	2.01 ± 1.39	0.00%	4.32 ± 5.12	0.00%	5.89E−11
P(v-a)CO_2_/C(a-v)O_2_ ratio	1.51 ± 7.47	47.09%	1.64 ± 4.29	49.28%	0.24 ± 20.09	11.59%	3.19E−01
PI	2.53 ± 1.46	0.00%	2.6 ± 1.45	0.00%	1.4 ± 1.15	0.00%	2.58E−38
Hb (g/dL)	108.21 ± 19.31	0.00%	108.94 ± 19.04	0.00%	96.47 ± 19.86	0.00%	5.17E−18
Balance (mL)	− 381.23 ± 1869.41	0.00%	− 366.59 ± 1665.24	0.00%	− 618.53 ± 3897.8	0.00%	3.28E−01

Consequently, the probability of an observation x_i_ categorized as the nth phenotype was

3 $${\text{ p}}\left( {{\text{z}} = {\text{n|}}x_{i} } \right) = {\raise0.7ex\hbox{${{\text{p}}\left( {{\text{z}} = {\text{n}}} \right)Normal\left( {\mu_{n} ,\sigma } \right)}$} \!\mathord{\left/ {\vphantom {{{\text{p}}\left( {{\text{z}} = {\text{n}}} \right)Normal\left( {\mu_{n} ,\sigma } \right)} {\mathop \sum \nolimits_{{{\text{l}} = 1}}^{5} {\text{p}}\left( {{\text{z}} = {\text{l}}} \right)Normal\left( {\mu_{l} ,\sigma } \right)}}}\right.\kern-\nulldelimiterspace} \!\lower0.7ex\hbox{${\mathop \sum \nolimits_{{{\text{l}} = 1}}^{5} {\text{p}}\left( {{\text{z}} = {\text{l}}} \right)Normal\left( {\mu_{l} ,\sigma } \right)}$}}$$

With an observation x_i_ of [33.0, 37.0, 26.57, 16.0, 118, 63.0, 98.0, 20.0, 34.0, 29.0, 498.0, 141.0, 51.0, 23.0, 92.0, 9.0, 13.0, 2.0, 5.0, 1.0, 97.0, − 2165.0] in the variable space as an example, we can calculate the five-class probabilities [0.034, 0.0085, 0.957, 0.00, 0.00052] with Eqs. (2) and (3). Taking the phenotype of the maximum probability implied that observation x_i_ was categorized as phenotype V.

**Fig. 1:**
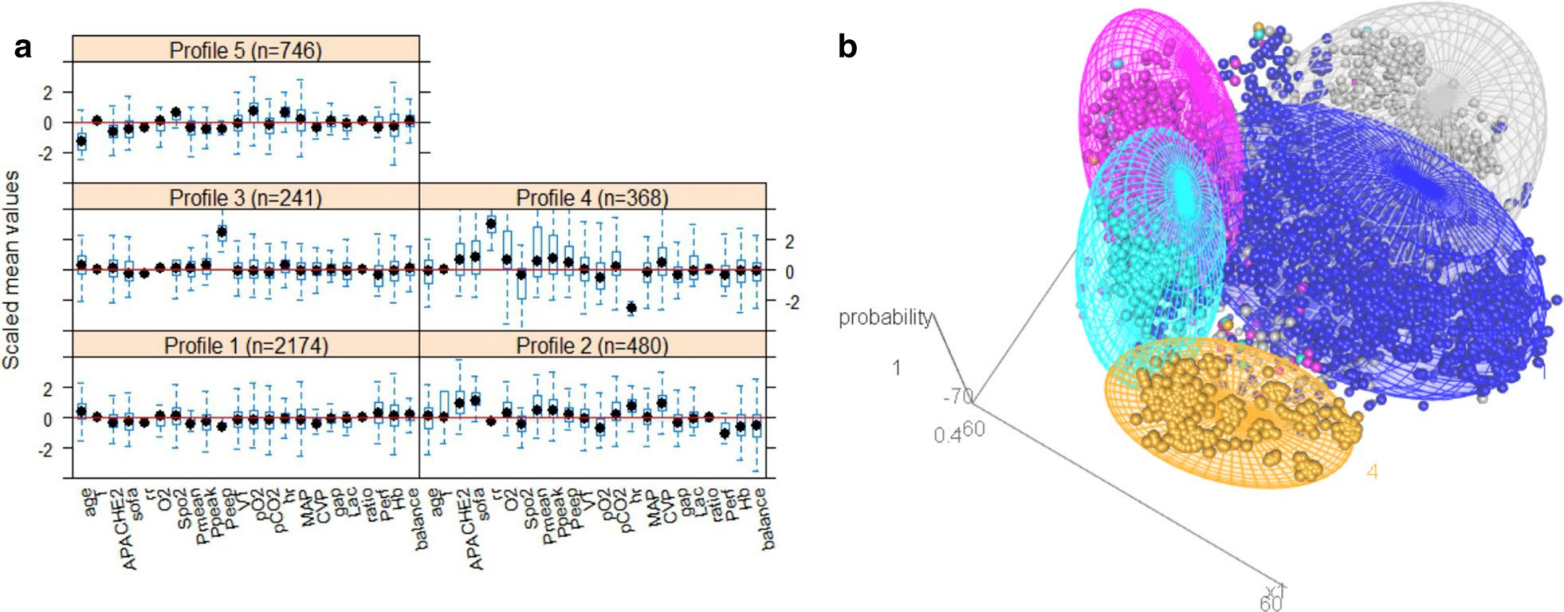
Variable distribution after normalization (**a**) and sample distribution in a 3-dimensional space {× 1, × 2, probability} (**b**) of the five profiles. Colors represent different profiles. Variable differences are closely related to profile divisions

As shown in Fig. 1a and Table 2, the variables were scaled for each phenotype. Broad differences were observed in the distributions of the scaled variables across phenotypes. Of the 22 variables measured, 18 were significantly different across phenotypes with *P* < 0.05. Several analyses were conducted to ensure that the phenotypes were not simply recapitulations of more traditional clinical groups. To that end, we first assessed the associations between the score variables and measures of illness severity, such as the APACHE II and SOFA scores. The mean APACHE II/SOFA scores for phenotypes II and IV were 23.40/11.45 and 21.31/9.79, respectively, which were significantly higher than those for phenotypes I, III and V (13.96/5.90, 16.64/6.78 and 11.31/5.01, respectively). For RR, FiO_2_, Pmean and Ppeak in terms of respiration, the means for phenotype IV were approximately 97.8, 48.9, 11.8, and 218, respectively; these values exceeded those of the other for phenotypes by 498%, 23%, 30.9% and 19%, respectively. Furthermore, for the HR in terms of circulation and perfusion, the differences across phenotypes IV and I, II, III, and V were also large. The mean for phenotype IV was 18.9, which was only 23.3%, 18.9%, 21.3% and 19.2% of that of the other four profiles. Phenotype III had a mean PEEP of 17.42, while the PEEP of phenotypes I, II, IV and IV was 5.74, 8.14, 10.21, and 5.9, respectively; the value for phenotype III exceeded those of the other phenotypes by 203%, 114%, 70.6%, and 195%, respectively. Phenotype V corresponded to an average age of 40.17 years, which was much lower than that of the other four phenotypes, which were 66.58, 58.54, 59.37 and 55.2 years.

**Table 2:** Characteristics of five mechanical ventilation phenotypes

Features (mean ± sd, missing ratio)	Profile I (n = 2174)	Profile II (n = 480)	Profile III (n = 241)	Profile IV (n = 368)	Profile V (n = 746)	*p* value
Age (years)	66.1 ± 11.87	58.36 ± 16.13	62.63 ± 14.54	55.85 ± 17.36	38.15 ± 11.84	0.00E+00
T (℃)	36.83 ± 0.51	37.19 ± 0.63	37.13 ± 0.57	37.07 ± 0.81	37.17 ± 0.51	2.74E−47
APACHE2	13.96 ± 4.97	23.4 ± 7.49	16.64 ± 6.32	21.31 ± 10.29	11.51 ± 5.05	1.69E−01
SOFA	5.9 ± 2.71	11.45 ± 2.75	6.78 ± 3.19	9.8 ± 5.04	5.01 ± 2.71	7.04E−01
RR (insp/min)	15.32 ± 1.98	17.6 ± 2.64	16.22 ± 2.22	18.92 ± 5.02	15.62 ± 2.21	5.21E−107
FiO_2_ (%)	37.25 ± 5.48	43.47 ± 7.44	40.74 ± 4.81	49 ± 19.29	36.88 ± 5.17	1.35E−13
SpO_2_ (%)	99.03 ± 1.12	98.19 ± 1.45	99.01 ± 1.27	96.45 ± 4.66	99.35 ± 0.88	6.17E−06
Pmean (cmH_2_O)	8.21 ± 1.13	10.6 ± 1.9	9.32 ± 1.5	11.86 ± 4.18	8.11 ± 1.14	1.16E−20
Ppeak (cmH_2_O)	17.1 ± 3.15	20.38 ± 3.45	19.25 ± 3.04	21.86 ± 6.47	16.72 ± 3.16	3.63E−08
PEEP (cmH_2_O)	5.75 ± 1.58	8.32 ± 2.8	17.52 ± 3.13	10.63 ± 6.2	5.94 ± 1.89	1.52E−39
VT (mL/B)	420.78 ± 63.22	426.28 ± 66.26	427.26 ± 64.11	439.69 ± 129.85	424.44 ± 68.16	7.09E−03
PO_2_ (mmHg)	133.22 ± 31.97	115.18 ± 31.35	137.1 ± 32.97	129.79 ± 61.95	159.41 ± 36.47	2.43E−44
PCO_2_ (mmHg)	38.26 ± 4.26	40.67 ± 4.61	38.56 ± 4.27	42.48 ± 9.31	37.49 ± 4.36	9.17E−02
HR (BPM)	81.52 ± 12.22	100.85 ± 14.83	88.45 ± 14.57	97.79 ± 20.56	98.29 ± 12.58	5.47E−03
MAP (mmHg)	88.86 ± 10.25	90.82 ± 9.54	89.1 ± 9.54	89.51 ± 17.33	92.29 ± 11.05	1.98E−10
CVP (mmHg)	7.56 ± 1.29	10.13 ± 2.14	8.43 ± 1.96	9.6 ± 2.97	7.47 ± 1.49	1.04E−06
Pv-aCO_2_ (mmHg)	5.55 ± 1.6	5.33 ± 2.27	5.96 ± 2.52	5.41 ± 2.57	5.82 ± 1.59	3.21E−03
Lac (mmol/L)	1.91 ± 1.05	2.37 ± 1.67	2.11 ± 1.35	3.82 ± 4.83	1.91 ± 1.17	5.48E−10
P(v-a)CO_2_/C(a-v)O_2_ ratio	1.39 ± 2.68	1.63 ± 1.93	1.87 ± 5.52	0.82 ± 15.05	1.84 ± 4.22	2.71E−01
PI	2.86 ± 1.34	1.52 ± 1.05	2.22 ± 1.2	2.49 ± 2.36	2.32 ± 1.17	6.06E−18
Hb (g/dL)	111.57 ± 18.25	100.22 ± 19.43	107.79 ± 17.35	108.39 ± 23.52	105.03 ± 18.77	7.76E−14
Balance (mL)	− 123.08 ± 1186.21	− 1613.92 ± 2873.5	− 225.94 ± 2316.81	− 691.79 ± 3158.67	− 300.57 ± 1288.97	3.34E−03

The means and standard deviations of the five phenotypes are shown in Table 2. To visualize the differences among all the phenotypes, t-SNE plots were used to reduce the dimensionality. Namely, nonlinear dimensionality reduction was performed in a 22-dimensional variable space to form 2-dimensional features ranging from [− 80, 70] to [− 80, 80]. Moreover, combined with the maximum probability from Eq. 3, a 3-dimensional space {x_1_, x_2_, probability} was thus formed. Therefore, as shown in Fig. 1b, we observed five distinct clusters that were formed in this 3-dimensional space, which are denoted in blue, pink, cyan, yellow, and gray and characterize the five respiratory phenotypes. This result indicated that there was strong separation in the likelihood of membership for patients assigned to a given phenotype compared with those assigned to other phenotypes. The number of patients assigned to phenotypes I, II, III, IV and V was 2174, 480, 241, 368, and 746, respectively, and the corresponding proportion of the total number of patients was 54.2%, 12.0%, 6.01%, 9.18% and 18.6%, respectively.

### Severity assessments and prognosis for the 28-day survival curve

**Fig. 2:**
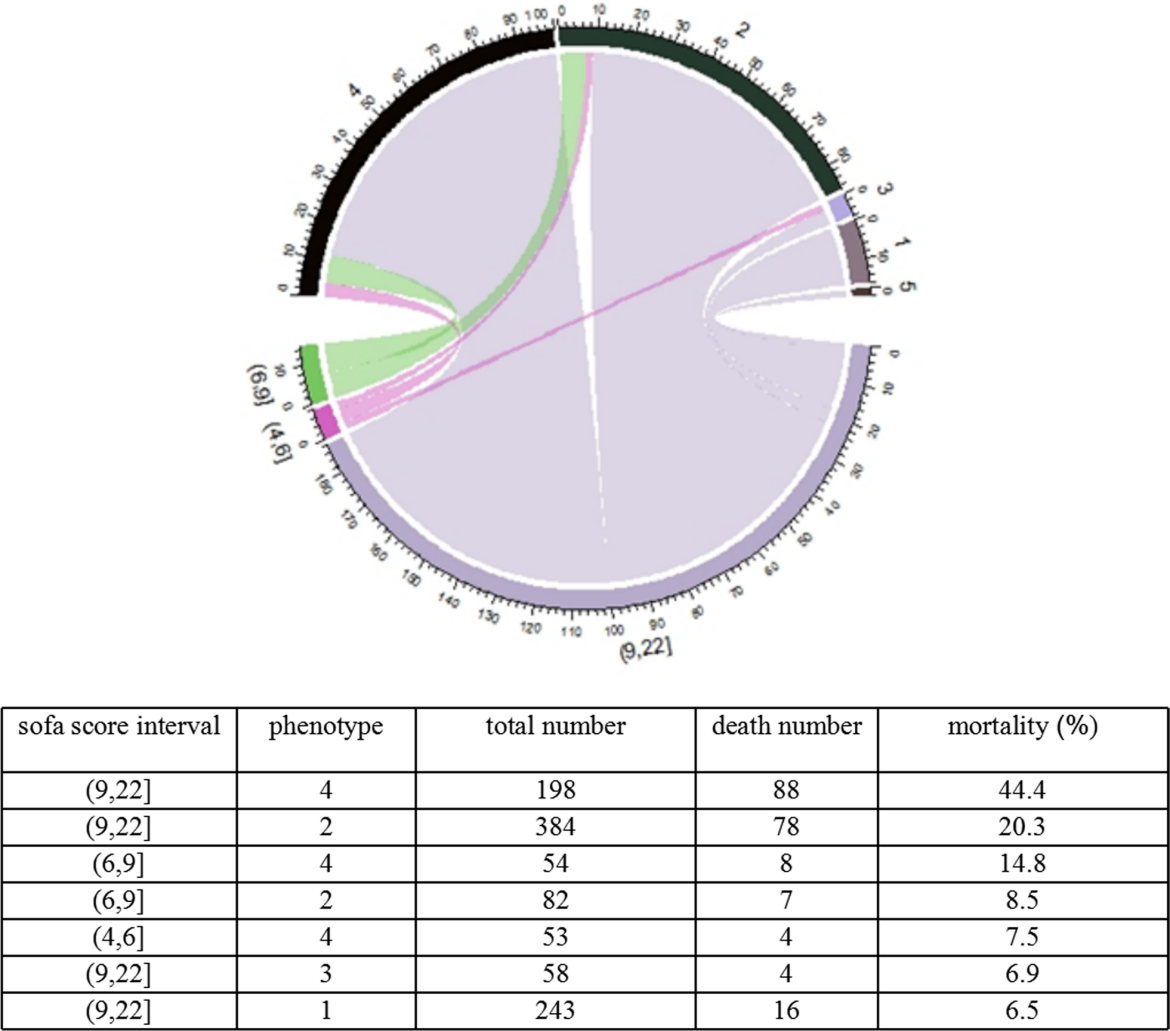
Relationships among the SOFA score segment, profile and number of deaths. In the top half of the gap in the figure, different colors and the outer circle numbers represent the deaths of different profiles. In the bottom half, different colors and the outer circle number intervals represent the deaths of different SOFA score segments. The details are shown in the table below

Since the APACHE II and SOFA values of phenotypes II and IV were significantly higher than those of the other phenotypes and the respiratory characteristics (RR, FiO_2_, Pmean, and Ppeak) and circulatory characteristics (HR) of phenotype IV were significantly different from those of the other four phenotypes, we used the SOFA score as an example by dividing it into four score intervals: [0, 4], (4, 6], (6, 9] and (9, 22]. We calculated the total number of patients and the mortality of each profile when the mortality rate of the four score intervals was more than 2%. As shown in Fig. 2, considering phenotype IV, which has the highest mortality rate, the number of patients in the SOFA score segment (9, 22] was 198, the number of deaths was 88, and the mortality rate was higher than 44%. The mortality of phenotype V in each score segment was less than 2%, so it was not included in Fig. 2.

**Fig. 3:**
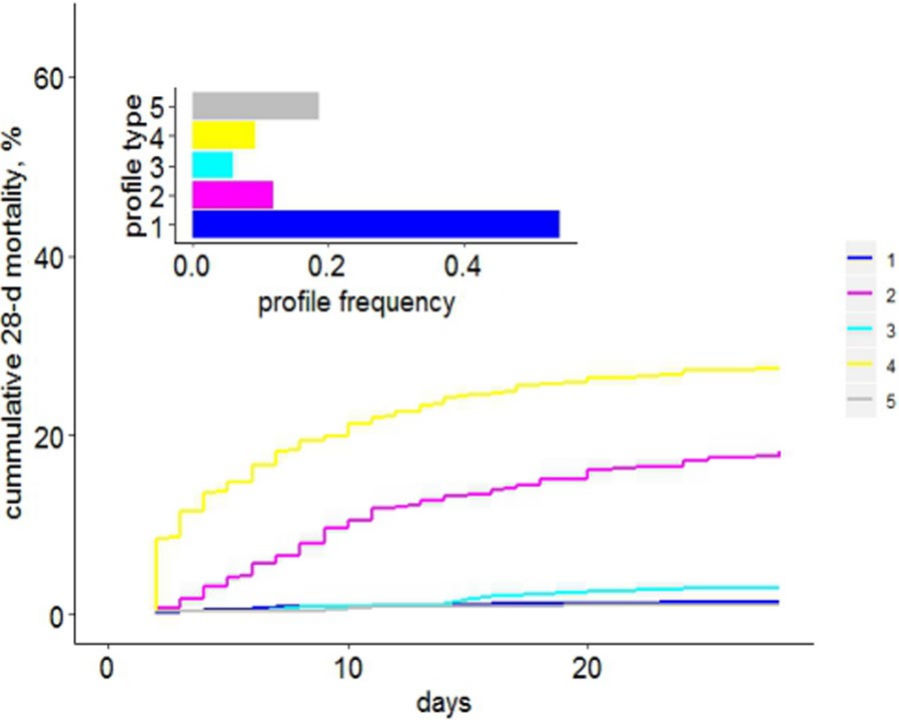
Proportion of patients of each profile and the corresponding 28 natural survival curves

Furthermore, the chord graph shown in Fig. 2 can effectively reflect the interactions among the SOFA score segment, section and the number of deaths. The upper and lower arcs of the graph correspond to the number of deaths in the section and SOFA score segment and interact with the two in the sphere with different colored surfaces. The number of deaths for phenotypes I, II, III, IV and IV was15, 4, 107, and 120, respectively, and the mortality rate was 6.1%, 1.6%, 43.4% and 48.8%, respectively. Cross-sections I, III, II and IV correspond to the upper halves of the arc denoted by the pink curved surface in a counterclockwise manner, as shown in Fig. 3. The proportions of individuals with phenotypes I, III and V among all cases were 54.2%, 6% and 18.6%, respectively, and the mortality rates for these three phenotypes were 1.23%, 1.40% and 1.59%, respectively, which were all smaller than 2.00%. The proportions of patients with phenotypes II and IV accounted for 12.0% and 9.2% of all patients, respectively, with mortality rates of 27.4% and 18.1%, respectively.

### Model stability verification

**Fig. 4:**
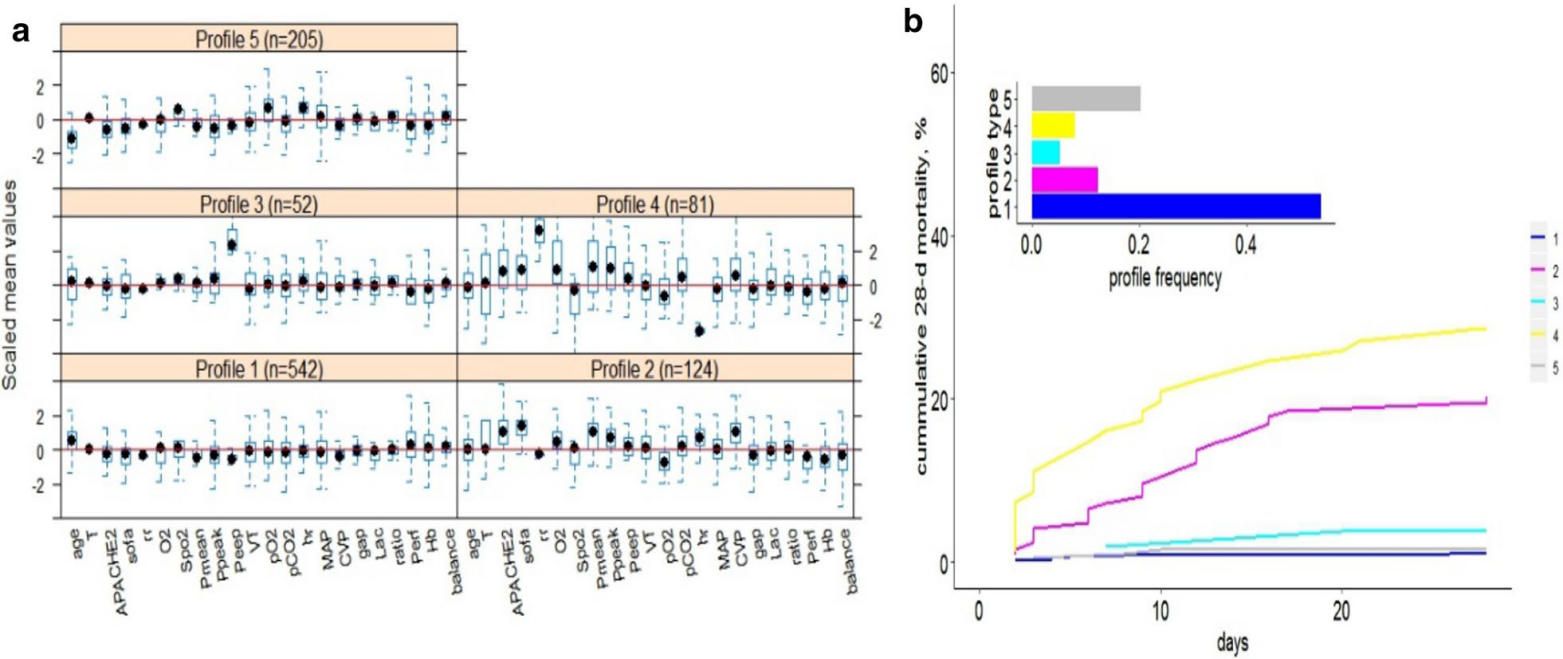
Characteristic distribution for each phenotype (**a**) and 28-day accumulative mortality curve (**b**) in the validation cohort

The validation cohort included 1004 patients, and the summary statistics are depicted in Table 3. Predictions of the phenotype distributions were generated for the validation cohort with the above model. This result was similar to that in the above training cohort. The number of patients assigned to phenotypes I, II, III, IV and V were 542, 124, 52, 81, and 205, respectively, and the corresponding proportions of the total number of patients were 54.0%, 12.4%, 5.18%, 8.07% and 20.4%, respectively (Table 4). Compared with the profile in Fig. 1a, that in Fig. 4 shows consistent differences in the biomarker patterns. To check the phenotype population stability, a metric (population stability index, PSI) was used to identify differences shifts in the population with the following formula:

4 $${\text{PSI }} = \sum {\left( {actual\% - expected\% } \right) \times {\text{ln}}\left( {\frac{actual\% }{{expected\% }}} \right)}$$

**Table 3:** Summary statistics of selected features in each group in the validation cohort

Features (mean ± sd, missing ratio)	Whole group (n = 1004)		Survivors (n = 945)		Nonsurvivors (n = 59)		*P* value
Age (years)	58.52 ± 16.41	0.00%	58.39 ± 16.37	0.00%	60.56 ± 17.02	0.00%	3.44E−01
T (℃)	37 ± 0.58	0.00%	37 ± 0.57	0.00%	37.02 ± 0.73	0.00%	8.62E−01
APACHE2	15.29 ± 7.35	11.65%	14.52 ± 6.65	12.06%	26.68 ± 7.91	5.08%	1.95E−16
SOFA	6.77 ± 3.82	11.65%	6.43 ± 3.52	11.85%	12.04 ± 4.35	8.47%	4.87E−13
RR (insp/min)	23.2 ± 24.24	0.00%	20.82 ± 19.93	0.00%	61.42 ± 46.05	0.00%	7.40E−09
FiO_2_ (%)	39.4 ± 9.58	0.00%	38.51 ± 7.98	0.00%	53.54 ± 18.24	0.00%	4.12E−08
SpO_2_ (%)	98.66 ± 2.17	0.00%	98.85 ± 1.42	0.00%	95.59 ± 6.2	0.00%	1.70E−04
Pmean (cmH_2_O)	8.96 ± 2.39	0.00%	8.75 ± 2.02	0.00%	12.39 ± 4.46	0.00%	5.25E−08
Ppeak (cmH_2_O)	18.19 ± 4.1	0.00%	17.95 ± 3.81	0.00%	21.98 ± 6.25	0.00%	1.00E−05
PEEP (cmH_2_O)	7.59 ± 5.48	0.00%	7.27 ± 4.94	40.32%	12.51 ± 9.61	0.00%	2.17E−03
VT (mL/B)	428.86 ± 71.1	0.00%	428.7 ± 71.04	0.00%	431.42 ± 72.65	0.00%	7.81E−01
PO_2_ (mmHg)	133.57 ± 37.13	0.00%	134.97 ± 36.33	0.00%	111.14 ± 42.61	0.00%	8.00E−05
PCO_2_ (mmHg)	38.78 ± 5.34	0.00%	38.58 ± 5.06	0.00%	41.88 ± 8.14	0.00%	3.14E−03
HR (BPM)	81.95 ± 24.68	0.00%	83.46 ± 22.62	0.00%	57.68 ± 39.6	0.00%	1.00E−05
MAP (mmHg)	89.96 ± 10.82	0.00%	90.08 ± 10.73	0.00%	88.07 ± 12.22	0.00%	2.21E−01
CVP (mmHg)	8.52 ± 2.42	41.73%	8.39 ± 2.29	43.81%	9.83 ± 3.16	8.47%	1.83E−03
Pv-aCO_2_ (mmHg)	5.77 ± 2.52	43.63%	5.79 ± 2.52	45.82%	5.48 ± 2.5	8.47%	3.85E−01
Lac (mmol/L)	2.17 ± 2.06	0.00%	1.99 ± 1.38	0.00%	5 ± 5.85	0.00%	2.20E−04
P(v-a)CO_2_/C(a-v)O_2_ ratio	1.68 ± 1.71	46.12%	1.68 ± 1.27	48.36%	1.68 ± 3.92	10.17%	9.98E−01
PI	2.54 ± 1.44	0.00%	2.58 ± 1.42	0.00%	1.81 ± 1.63	0.00%	7.70E−04
Hb (g/dL)	108.47 ± 19.21	0.00%	108.63 ± 18.82	0.00%	105.93 ± 24.61	0.00%	4.11E−01
Balance (mL)	− 403.72 ± 1926.05	0.00%	− 388.05 ± 1840	0.00%	− 654.73 ± 2996.65	0.00%	5.02E−01

**Table 4:** Characteristics of five mechanical ventilation phenotypes in the validation cohort

Features (mean ± sd, missing ratio)	Profile I (n = 542)	Profile II (n = 124)	Profile III (n = 52)	Profile IV (n = 81)	Profile V (n = 205)	*P* value
Age (years)	66.58 ± 11.37	58.54 ± 15.44	59.37 ± 17.11	55.2 ± 17.49	40.17 ± 10.98	9.39E−102
T (℃)	36.83 ± 0.49	37.16 ± 0.64	37 ± 0.49	37.01 ± 0.98	37.19 ± 0.48	4.9527E−13
APACHE2	14.11 ± 4.94	23.5 ± 7.21	14.79 ± 4.64	22.4 ± 10.97	11.54 ± 4.96	0.11487362
SOFA	5.85 ± 2.6	11.84 ± 2.84	6.14 ± 2.93	9.85 ± 4.5	5.37 ± 2.72	0.6420805
RR (insp/min)	15.53 ± 2.57	17.08 ± 2.58	16.04 ± 2.1	20.14 ± 5.07	15.62 ± 1.89	4.4237E−22
FiO_2_ (%)	36.79 ± 5.47	43.97 ± 7.43	41.13 ± 4.93	51.02 ± 17.19	36.37 ± 5.76	0.00019347
SpO_2_ (%)	98.99 ± 1.14	98.26 ± 1.41	99.19 ± 1.12	96.16 ± 5.98	99.39 ± 0.87	0.21325563
Pmean (cmH_2_O)	8.26 ± 1.11	10.73 ± 1.91	9.19 ± 1.52	11.86 ± 3.76	8.16 ± 1.1	0.00017063
Ppeak (cmH_2_O)	17.29 ± 3.41	20.92 ± 3.42	19.25 ± 3	21.91 ± 6.08	16.75 ± 3.16	0.17203648
PEEP (cmH_2_O)	5.74 ± 1.5	8.14 ± 3.04	17.42 ± 2.89	10.21 ± 6.78	5.9 ± 1.56	8.4678E−08
VT (mL/B)	420.85 ± 63.81	436.2 ± 69.98	419.73 ± 68.94	423.27 ± 104.77	421.87 ± 65.94	0.92926968
PO_2_ (mmHg)	130.76 ± 32.35	113.85 ± 35.64	138 ± 27.8	130.62 ± 59.47	157.53 ± 35.2	7.0463E−16
PCO_2_ (mmHg)	38.2 ± 4.32	40.21 ± 5.02	38.04 ± 4.97	43.06 ± 10.13	37.61 ± 4.08	0.31930209
HR (BPM)	81.53 ± 12.37	100.91 ± 14.87	86.54 ± 15.16	97.48 ± 22.85	99.1 ± 11.71	0.53986379
MAP (mmHg)	88.51 ± 10.08	90.02 ± 8.93	89.15 ± 10.57	87.25 ± 19.48	91.58 ± 11.32	0.01042881
CVP (mmHg)	7.55 ± 1.26	10.07 ± 2.21	8.18 ± 1.28	9.46 ± 2.87	7.61 ± 1.45	0.01687499
Pv-aCO_2_ (mmHg)	5.5 ± 1.55	5.29 ± 2.49	5.68 ± 1.58	5.44 ± 2.57	5.65 ± 1.78	0.37027728
Lac (mmol/L)	1.87 ± 1.03	2.63 ± 1.84	2 ± 1.24	4.11 ± 5.1	1.89 ± 1.1	0.0028773
P(v-a)CO_2_/C(a-v)O_2_ ratio	1.51 ± 0.88	1.52 ± 0.65	1.56 ± 1.13	1.54 ± 1.55	1.61 ± 0.62	0.20245812
PI	2.88 ± 1.27	1.64 ± 1.05	2.15 ± 1.02	2.47 ± 2.35	2.36 ± 1.19	4.9826E−06
Hb (g/dL)	110.55 ± 18.13	101.19 ± 17.9	103.25 ± 18.67	105.81 ± 22.91	104.63 ± 18.31	0.000066705
Balance (mL)	− 103.08 ± 995.45	− 1639.78 ± 3940.19	− 379.04 ± 1632.88	− 388.62 ± 2459.24	− 233.08 ± 1210.63	0.49045364

Additionally, we used the PSI to measure 28-day mortality by phenotype in two datasets. The number of deaths at 28 days were 2 (1.11%), 23 (20.2%), 6 (3.85%), 25 (28.4%) and 3 (1.46%). Then, we calculated the PSI for the phenotype populations and mortality across the two datasets. The final values were 0.0045 and 0.007, which indicated an insignificant difference in both the population size and mortality rate between the validation cohort and study cohort.

### External verification results

We used MIMIC III cohort for further verification. To minimize the impact of database differences, we finally chose 15 variables and divided them into four groups: demographics (age and temperature (T)) and scoring features (SOFA score and SAPSII score), respiratory features (Pmean, Ppeak, VT, SpO_2_%, RR, pO_2_ and PEEP), circulation and perfusion features (Hb, HR and MAP) and residual features (fluid balance).

**Fig. 5:**
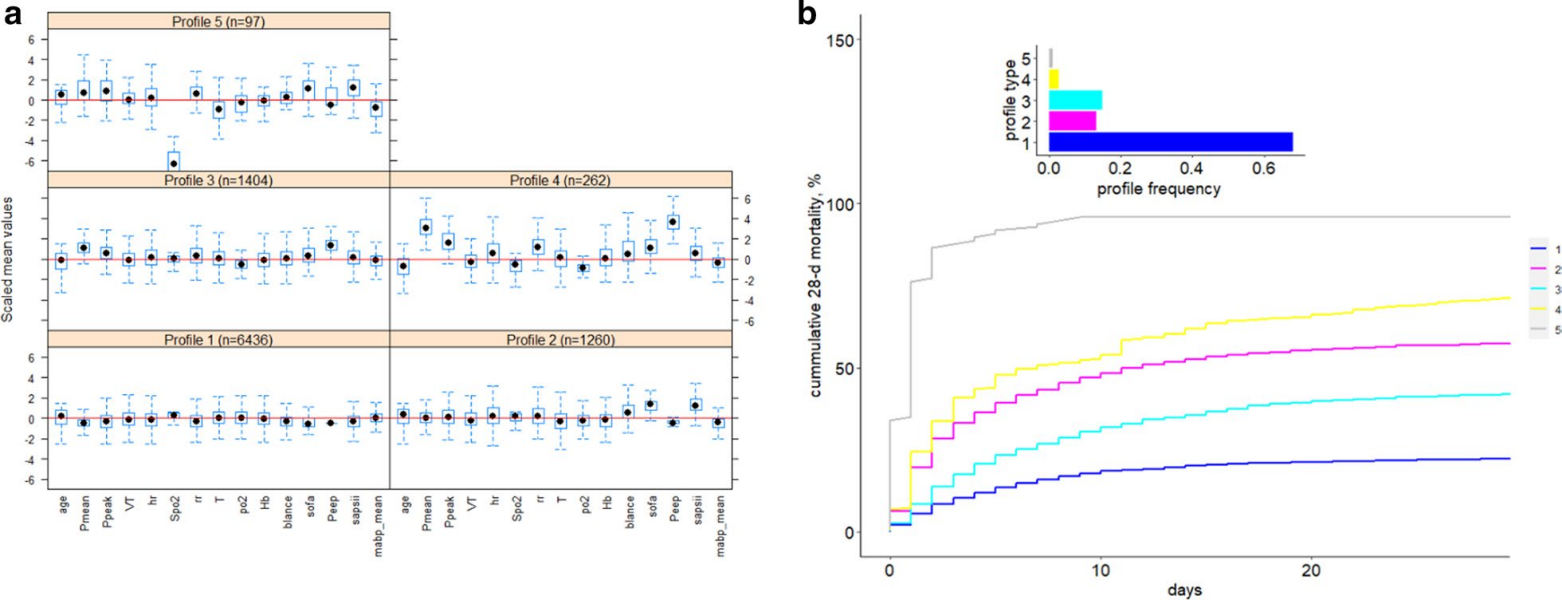
Characteristic distribution for each phenotype (**a**) and 28-day accumulative mortality curve (**b)** in the MIMIC III validation cohort

The clustering results may not be completely consistent with the data of Peking Union Medical College Hospital; however, the same five profiles were generated as for the Peking Union Medical College Hospital cohort, as displayed in Fig. 5. The SpO_2_ of Profile 5 was significantly lower than those of the other profiles, the PEEP values of Profiles 3 and 4 were higher than those of Profiles 1, 2 and 5, and the Pmean of Profile 4 was obviously different from those of the other profiles. While given the differences between databases in ethnic composition, recording methods and data types, the verification results demonstrated that our phenotyping method has generalizability and the model is stable to divide patients undergoing MV into five different subtypes.

## Discussion

In this retrospective ICU MV patient study, we used machine learning methods to obtain five ventilator-related phenotypes on the day of ICU admission based on the patient's general data, ventilator parameters and partially monitored circulating perfusion indicators, as well as the in-and-out balance (residual characteristics) within 24 h of the day. For these five types of respiratory phenotypes, we used a chord diagram to suggest that the interaction among the SOFA score, profile, and death toll showed that Profiles IV and II had the highest mortality. Survival curve analysis revealed that the 28-day survival rate of Profile IV and Profile II was significantly lower than the those of the other three profiles. We constructed five ventilator-related phenotypes with mathematical expressions using a potential profile approach and used validation data sets to predict and validate the existence of the five phenotypes. Additionally, the experiments on the MIMIC III cohort confirmed these phenotypes. The implementation of this study facilitates the identification and treatment of these mechanically ventilated patients in clinical practice, improving patient survival and improving prognosis.

Inappropriate ventilation strategies during MV result in lung injury, and the mechanisms include high airway pressures or tidal volumes resulting in lung pressure/volume injury. Excessively low expiratory lung volumes or atelectasis lead to repeated opening and collapsing of the terminal lung unit. In addition, in patients undergoing MV, even without anatomical changes in the lung tissue, the effects of various forces can induce the release of pro-inflammatory cytokines and the recruitment of white blood cells, and the initiation of local inflammatory processes is called biological injury [9]. The first three injuries are considered to be mechanical injuries caused by mechanical factors, while the remaining injuries are caused by mechanical damage caused by secondary damage involving inflammatory cells and inflammatory mediators. Therefore, information on how to adjust and control MV is clinically important. Previous studies have shown that low tidal volumes, high PEEPs and proper control of the plateau pressure are important for resolving the current MV problem [10, 11]. Even simply limiting the VT and plateau pressure is not completely safe. On the other hand, contradictory situations in which various protective strategies are contradictory may also occur in clinical practice. For example, increasing the PEEP will cause a corresponding increase in the plateau pressure when volume control mode, and often, patients with more severe lesions and poorer lung compliance will reach the an acceptable maximum limit of the plateau pressure (often known as 40 cmH_2_O) at a PEEP lower than the required level. Therefore, we urgently need to use new methods to examine traditional clinical problems.

Through analysis, we found that MV patients could be divided into five subtypes. For example, in MV patients of Peking Union Medical College Hospital, Profile IV showed a high mean airway pressure, a high peak airway pressure, a fast respiratory rate and a slow heart rate. Profile III showed a high pure PEEP. Based on the survival analysis, we can clearly see that the mortality rate was the highest for Profile IV compared with the other profiles. Namely, the prognosis of patients who show improved oxygenation by increasing the airway pressure is worse than that of patients who show improved oxygenation by increasing the PEEP without increasing the airway pressure. The occurrence of VILI depends on the interaction of the ventilator and the lungs, i.e., the pressure, volume, flow rate and frequency of the ventilator applied to the lungs and the reactivity of the lung parenchyma. The pressure generated in the lung tissue when MV is used to expand the lung is called stress, which is reflected as transpulmonary pressure. The driving pressure may better reflect the actual ventilation state in terms of the MV phenotype. Namely, with the same tidal volume, the larger the amount of functional residual gas there is, the smaller the strain generated, the smaller the probability of the occurrence of VILI, and the better the prognosis of the patient. In 2015, Amato et al. proposed that for patients with ARDS undergoing MV, the VT, PEEP, and driving pressure (DP) are independent factors [12]. Among these factors, the DP level had the strongest correlation with the survival rate of ARDS patients. The study also suggested that the PEEP and small VTs only play roles in lung protection when the DP is reduced. Neto et al. [13] found that DP is the only factor strongly correlated with postoperative pulmonary complications by performing a high-quality meta-analysis of MV risk [14]. In another study of ARDS, it was found that a DP higher than 14 cmH_2_O significantly affects the prognosis of patients [13].Our study using big data also demonstrates the role of DP in determining the phenotype of patients undergoing MV. In other words, it is only used in patients with good recruitibility to increase the PEEP, which is beneficial, and in patients with poor recruitibility, increasing the PEEP causes excessive expansion, which is harmful [11, 15].

Phenotype V represents a younger but higher-scoring patient, and this phenotype may become an independent phenotype, probably due to lung compliance. The parameters of MV did not appear to have an effect on these aspects of the patients, and the prognosis of the patients were good. Phenotype II is different from phenotype III, as the patients classified as phenotype II have higher scores, but they are not considerably different in terms of age. These patients had poor organ function which may not be only reflected from the lung-related variables, so these patients may form an independent group as phenotype II. The last group is phenotype I, which represents the majority of clinical patients and the most homogeneous group. We further analyzed the departments in which the patients were admitted and found that phenotypes I, III, and V were from surgery, and phenotypes II and IV were from internal medicine.

This study also has some limitations. First, although it was a big data study, there was a training set combined with a verification set to validate five phenotypes. We used the data from the MIMIC III cohort to demonstrate our findings. However, this model still needs to be clinically verified by a prospective study. Second, the study included general information, ventilator parameters and circulation and perfusion indicators, as well as the balance of input and output (residual characteristics) within 24 h of the day, which take into account some of the indicators commonly considered in clinical use of MV. It is not known whether it is necessary to include all the indicators that can be obtained and recorded in the clinic or to combine some indicators. Third, many of the parameters included in this study are related to clinical decision making. For example, PEEP, VT and other parameters are more decisions of the clinician than the patient's own respiratory mechanics. Therefore, phenotypic analysis in different institutions may conclude different results. The current conclusion of this study requires further multicenter validation if possible.

## Conclusions

In this retrospective analysis, respiratory and circulatory data were obtained from patients undergoing MV from two databases. Five ventilator-related phenotypes were obtained, and from these five phenotypes, Profiles II and IV were both related to mortality, and Profile IV was more significant in higher mortality caused by mechanical ventilation parameters. These five new identified phenotypes of critically ill patients undergoing MV may be helpful in the future identification and interpretation of clinically high-risk patients.

## Data Availability

Not applicable.
